# Rewiring autophagy to target cutaneous wound healing: investigation of molecular mechanisms to therapeutic interventions

**DOI:** 10.1007/s11033-026-12938-w

**Published:** 2026-10-16

**Authors:** Keerthan Nayak, Ishani Banerjee, Fahika Nazerulla, Aditi Valame, Manash Kumar Paul, Sujit Kumar Bhutia, Deblina Basu, Subhadip Mukhopadhyay

**Affiliations:** 1 Department of Biotechnology, Manipal School of Life Sciences, Manipal Academy of Higher Education, Manipal, 576104 Karnataka India; 2 Department of Radiation Biology and Toxicology, Manipal School of Life Sciences, Manipal Academy of Higher Education, Manipal, 576104 Karnataka India; 3 Department of Life Science, National Institute of Technology Rourkela, Rourkela, 769008 Odisha India; 4 Department of Microbiology, Grossman School of Medicine, New York University (NYU) Langone Health, New York, 10016 New York United States of America

**Keywords:** Autophagy, Wound healing, Chronic wounds, Inflammation, Phytochemicals, Biomaterials, Pharmacological modulators

## Abstract

Rewiring of pathways regulating inflammation, metabolism, and regeneration must occur in a coordinated fashion during wound healing; however, this orchestration gets disrupted in chronic and diabetic wounds. Autophagy is a conserved lysosome-dependent pathway that controls the integration of nutrient homeostasis, adaptation to stresses, immune regulation, and cell remodelling during wound repair. Here we summarize the pivotal role of autophagy in the different crucial steps of wound healing. An open wound often attracts pathogens, leading to infection and inflammation. During this inflammation stage, autophagy mediates signalling from the pattern recognition receptors, generates cytokines, manoeuvres the defence against infection, and polarizes macrophage. During proliferation stage, autophagy promotes keratinocyte migration, fibroblast function, endothelial cell survival, angiogenesis, while minimizing oxidative injury and apoptosis. During the remodelling stage, autophagy participates in the remodelling of the extracellular matrix, collagen maturation, and scar formation. However, these processes are context-dependent; while insufficient autophagy prevents adaptation and regeneration, we see excessive autophagy may contribute to pathological conditions associated with chronic wounds. The analysis also considers new therapeutic approaches including mTOR and AMPK inhibitors, phytochemicals, exosomes from mesenchymal stem cells, and the use of biomaterials for the delivery of drugs. Although they improve re-epithelization, vascularization, and matrix organization in preclinical models, the clinical application of these approaches is hampered by the lack of specificity of the pathway, poor stability and penetrability of drugs, off-target effects, and a lack of clinical trials in humans.

**Supplementary Information:**

The online version contains supplementary material available at https://doi.org/10.1007/s11033-026-12938-w.

## Introduction

Wound healing is an essential biological process that preserves skin integrity after injury, involving a complex and highly coordinated interplay of physiological mechanisms [1]. In the skin, this process is divided into hemostasis, inflammation, proliferation, and remodelling. All of which must occur in the correct order and with appropriate intensity to restore function efficiently [2]. When this balance is disturbed, healing can become delayed or dysregulated, leading to chronic non-healing wounds. An estimated 2.21 per 1000 people of the global population are affected by chronic wounds, which typically last 12 to 13 months, recurring in as many as 60% to 70% of patients, and can result in diminished function and a lower quality of life [3–5]. The process of wound healing is evolutionarily conserved, and depends on the interaction of immune cells, fibroblasts, keratinocytes, endothelial cells, extracellular matrix components, and signaling mediators at the injury site to bring back the equilibrium and normal functioning at the site of damage [6]. Living organisms have devised strategies to reverse or fix the problem caused by conflict or accident. In lower organisms, the process of repairing the cells and tissues is almost same as the pre-wound condition. However, in higher mammals, the same repair leaves a scar at the site of the wound [7].

Hemostasis occurs first, limiting blood loss and generating a provisional matrix that supports cell migration, followed by inflammation, which clears debris and pathogens and prepares the wound bed for tissue repair. Vascular constriction occurs at the site of injury, followed by platelet activation upon exposure to subendothelial collagen via Glycoprotein Ia-IIa(GPIa-IIa) and Glycoprotein Ib-IX-V(GPIb-IX-V), which triggers the formation of platelet plugs at wound site. Activated platelets initiate the coagulation cascade, forming a fibrin-rich clot like network that seals the wound and supports cell migration [8, 9]. The coagulation cascade network is activated via intrinsic or extrinsic pathways, generating thrombin that converts fibrinogen to crosslinked fibrin, forming a hemostatic clot that also serves as a temporary extracellular matrix [9]. Hemostasis is followed by the inflammatory phase, which establishes a microenvironment conducive to tissue repair through the clearance of pathogens, damaged tissue, and debris [6]. Increased vascular permeability facilitates the recruitment of innate immune cells in response to damage-associated molecular patterns (DAMPs) and pro-inflammatory cytokines. Neutrophils first develop at the wound site, mediating microbial clearance via reactive oxygen species (ROS), cytotoxic granules, neutrophil extracellular trap (NET) formation, and cytokine release thereby amplifying inflammation by producing tumor necrosis factor alpha (TNF- α), interleukin 1 and 6 (IL-1, IL-6), and C-X-C motif chemokine ligands −8, −2 (CXCL8, CXCL2) along with monocyte chemoattractant protein-1 (MCP-1). These cytokines/chemokines further recruit more monocytes/macrophages at the injury site [10, 11]. Macrophages exhibit functional plasticity, with pro-inflammatory macrophages M1 phenotypes promoting inflammation and pathogen clearance, while anti-inflammatory macrophage M2 phenotypes drive resolution and tissue repair through anti-inflammatory signalling [2]. Dysregulation of this phase can result in persistent inflammation and impaired wound healing. Wound healing moves into the proliferation phase within about three days, marked by the buildup of granulation tissue [12]. Growth factors like platelet derived growth factor (PDGF) and transforming growth factor beta (TGF-β) draw fibroblasts into the wound, where they rapidly multiply and lay down a new extracellular matrix of proteoglycans, fibronectin, and Type III collagen in place of the fibrin clot [12, 13]. Meanwhile, vascular endothelial growth factor (VEGF) drives endothelial cells to form new blood vessels that feed the healing tissue [14], and keratinocytes migrate across the wound bed to reseal the skin barrier [10, 14]. Remodelling follows as the final, often year-long stage, during which the extracellular matrix (ECM) is reorganized and type III collagen is gradually replaced by stronger type I collagen, restoring tensile strength and the resulting scar rarely regains more than 70–80% of the original tissue's strength [10, 13]. This balance of matrix synthesis and breakdown is regulated largely by matrix metalloproteinases [15], while myofibroblasts, fibroblasts, and endothelial cells are gradually cleared through apoptosis [16], leaving behind a mature, avascular scar with reduced vascularity and cell density [9, 12].

Autophagy has emerged as an important regulator of these repair events, integrating nutrient sensing, stress adaptation, immune signaling, and cellular remodelling as the wound heals [12, 17]. As a conserved lysosome-dependent degradation pathway, it clears damaged organelles, protein aggregates, and intracellular pathogens while recycling cellular components to sustain homeostasis under metabolic or environmental stress [15, 18, 19]. Because the wound microenvironment is marked by hypoxia, oxidative stress, inflammation, and nutrient limitation, these conditions readily activate autophagic responses and shape healing outcomes [20–22]. Rather than a simple housekeeping process, autophagy therefore acts across every stage of repair- inflammation, proliferation, and remodelling influencing immune regulation, cell survival, matrix turnover, and tissue regeneration [9, 17].

Pharmacological agents such as rapamycin and metformin, phytochemicals including resveratrol, epigallocatechin gallate, and curcumin, as well as mesenchymal stem cell-derived exosomes and biomaterial-based delivery systems, have been investigated for their ability to regulate autophagy and improve wound healing outcomes. Preclinical studies indicate that these approaches can improve re-epithelialization, vascularization, and matrix organization, pointing to real therapeutic potential [23–28]. Despite this progress, translation into clinical practice remains limited by several factors, including poor drug solubility, limited skin penetration, instability, off-target effects, and the scarcity of robust clinical trials [17, 29, 30]. Therefore, a more precise understanding of autophagy in specific wound contexts is necessary to guide therapeutic development [17, 18].

This review summarizes the role of autophagy in cutaneous wound healing, with an emphasis on its functions across the different stages of repair, the molecular mechanisms involved, and the therapeutic potential of autophagy-targeting interventions [9, 19]. It also highlights the current limitations and future directions for translating autophagy-based strategies into clinically useful treatments for acute and chronic wounds [17].

## Physical significance of autophagy

Autophagy is a cellular degradation and recycling pathway whose molecular pathway is conserved across the eukaryotes [31]. Autophagy acts as a self-renewal mechanism [9], which enables the cell to recycle cellular waste and components through lysosome [15] and help in cellular and genomic stability [18]**.** This process plays an important role in maintaining homeostasis of the cell and protects it from death at the time of stress and lack of nutrients [15, 19].

Autophagy is classified into different forms based on cargo delivery patterns and physiological functions. Macroautophagy, is the most extensively studied form and is commonly referred to as autophagy. In this process, a phagophore is initiated, which expands to sequester cytoplasmic cargo and is followed by the formation of a double membrane bound vesicle called autophagosome. This then fuses with a lysosome (formation of autolysosome) where the engulfed materials are broken, by the action of hydrolytic enzyme from lysosome [15]. Microautophagy and chaperone-mediated autophagy (CMA) are the other forms. Similarly, autophagy can be classified based on the specific component of cell getting degraded such as xenophagy, mitophagy, ER-phagy, lipophagy, etc. [18, 19].

Autophagy is regulated by cellular nutrient and energy sensing pathways, with mammalian target of rapamycin complex 1 (mTORC1) acting as the principal negative regulator and AMP-activated protein kinase (AMPK) serving as the primary positive regulator. Under nutrient-rich conditions, activation of the phosphatidylinositol-3-kinase PI3K–AKT, also called protein kinase B pathway stimulates mTORC1, which suppresses autophagy by inhibiting the unc-51 like autophagy activating kinase 1 (ULK1) complex. In contrast, nutrient deprivation, hypoxia, oxidative stress, and ATP depletion activate AMPK, which inhibits mTORC1 while directly phosphorylating ULK1 and Beclin1 to initiate autophagy [20, 21]. These stress conditions are frequently encountered within the wound microenvironment, making autophagy an important adaptive response during tissue repair.

**Fig. 1:**
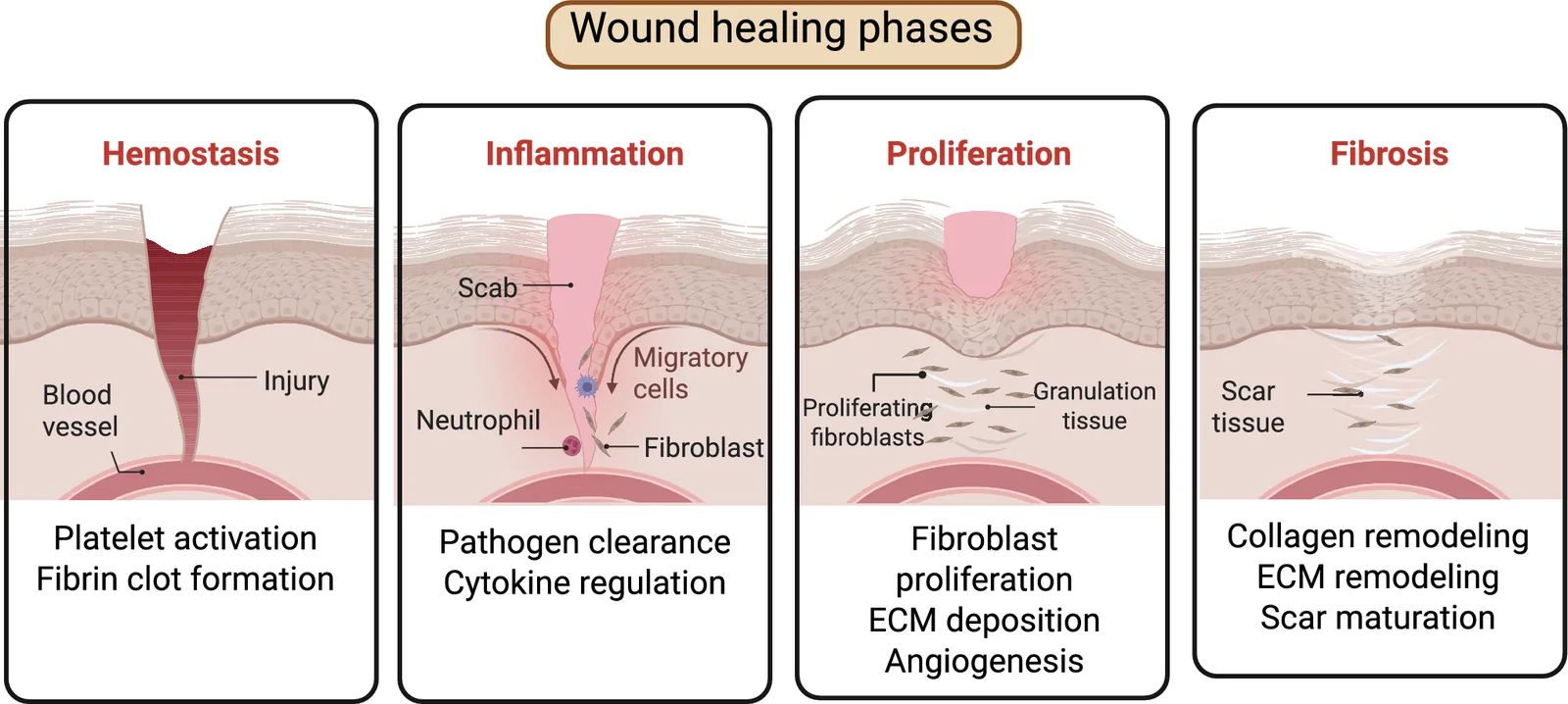
Different stages of wound healing. Wound healing involves multiple phases of coordination led by immune homeostasis, pathogen clearance, supporting keratinocyte migration and fibroblast proliferation, and facilitating extracellular matrix (ECM) remodelling. Created in BioRender. Rai, P. (2026) https://BioRender.com/rqu1ftp

Autophagosome formation occurs through four sequential stages: initiation, nucleation, elongation, and lysosomal fusion. Activation of the ULK1 complex (ULK1–ATG13–ATG101–FIP200) recruits the class III PI3K complex (VPS34–Beclin1–ATG14), generating phosphatidylinositol-3-phosphate (PI3P) at the phagophore assembly site. PI3P subsequently recruits downstream ATG proteins, including the ATG5–ATG12–ATG16L1 conjugation system, which promotes microtubule-associated protein 1A/1B-light chain 3 (LC3) lipidation (LC3-I to LC3-II) and autophagosome membrane expansion [22, 32]. The mature autophagosome finally fuses with lysosomes through a small GTPase known as RAB7- and soluble N -ethylmaleimide-sensitive factor attachment protein receptors (SNARE)-dependent mechanisms, enabling degradation and recycling of intracellular cargo [33, 34]. By removing damaged organelles, protein aggregates, and intracellular pathogens while recycling metabolic substrates, autophagy preserves cellular homeostasis under stress. These functions are fundamental to wound repair, where autophagy regulates inflammation, oxidative stress, angiogenesis, extracellular matrix remodelling, and tissue regeneration [9, 17] (Fig. 1).

## Autophagy and wound healing

### Autophagy in the initiation of inflammation and innate immune activation

Injury to the tissue in organisms initiates the immune system, which plays an important role in starting repair [35]. When the damage occurs, DAMPs are released by the dying/damaged cells along with PAMPs derived from invading microorganisms which initiate inflammation, essential for the defense against pathogen. The signals from the wound site are recognized by pathogen recognition receptors (PRRs), such as toll-like receptors (TLRs), which activate the inflammatory cascade. This stimulus activates tissue resident macrophages, and subsequently other immune cells are recruited at the site of injury [36, 37].

Upon tissue damage, activation of inflammatory mediators and PRR signaling stimulates autophagy, enhancing antimicrobial defense and modulating the inflammatory response. For example, autophagy related protein ATG16L1 is upregulated after microbial sensing by PRRs particularly NOD1 and NOD2 of the NOD-like receptor (NLR) family, which promotes the formation of autophagosomes around microbes for further degradation [38]. Studies in macrophage cell lines show that TLRs can promote autophagy after lipopolysaccharide (LPS) treatment, by stabilizing the key initiator protein Beclin-1 [39]. TLR2/TLR6 and TLR4 signaling mediates the prevention of excess inflammation and balances the immune response while TLR2 activation in kidney tubular epithelial cells showed enhanced autophagy during kidney injury [40]. Moreover, studies in UV-induced skin injury have shown that autophagy in macrophages facilitates macrophage differentiation and improves healing [41].

### Role of autophagy during inflammatory phase

Inflammation is the first response which plays a key role in directing regeneration and reparative function of the tissue [42]. During the inflammatory phase, many small molecules (cytokines and chemokines) are released, resulting in recruitment of neutrophils (Fig. 1). This further amplifies the inflammation by releasing cytokines like IL-1β, IL-6, TNF-α [43]. During the injury, activation of nuclear factor kappa-light-chain-enhancer of activated B cells (NF-κB) signaling and TNF mediated inflammatory pathway induces autophagy in keratinocytes. This induces CCL2 expression, which promotes the recruitment of macrophages, neutrophils, and mast cells to the wound site. CCL2 also acts as a potent chemoattractant for circulating monocytes, facilitating their migration and infiltration into injured tissues [6]. Beyond this recruitment role, autophagy also helps to regulate inflammation by controlling the secretion of inflammatory mediators. This activation of autophagy in macrophages increases the release of IL-1β and high mobility group box 1 (HMGB1) through the process of secretory autophagy [44], which may promote useful inflammatory signaling during repair [45]. In the inflammatory phase, autophagy modulates macrophage polarization and drives the switch from pro-inflammatory M1 state to the pro-repair M2 state [46, 47]. However, this switch can work adversely sometimes as demonstrated by [48]. This study shed light on advanced glycation end-products which stimulated autophagy and subsequently M1 polarization leading to impaired cutaneous wound healing via Interferon Regulatory Factor 8 (IRF8) activation. CCL6 has also been described to promote wound healing by increasing M2 macrophage levels through inhibition of macrophage autophagy in mouse skin wounds [47].

Outside of its role in immune signaling, autophagy is involved in eliminating invading microbes during the inflammatory stage. Autophagy receptors spot ubiquitinated pathogens and transport them for degradation [9].

### Role of autophagy during the proliferative phase

**Fig. 2:**
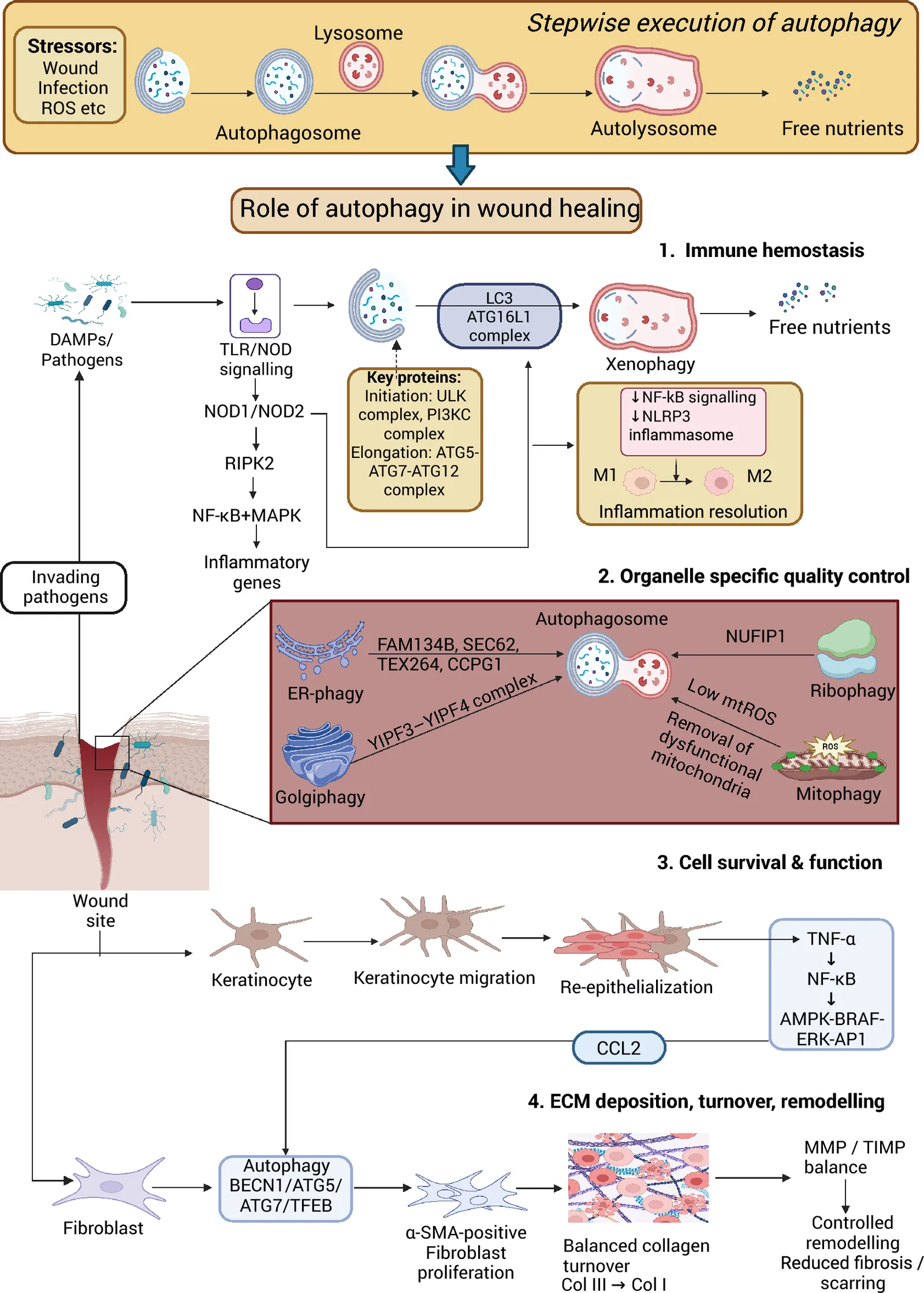
Role of autophagy in wound healing: Stress signals such as wound, infection, ROS activate critical pathways leading to autophagy induction. Autophagy coordinates immune defense, organelle quality control and tissue repair during wound healing. Step-1: Autophagy mediates the immune homeostasis of the wound site via TLR and NOD family of receptors. NOD1/2 recognizes the pathogens through the RIPK2 pathways thereby activating the NF-κB /MAPK pathway, which can control M1/M2 macrophage polarization. Parallelly, NOD1/2 can also recruit LC3/ATG16L1 proteins promoting lysosomal clearance through xenophagy. This activation of either the inflammatory genes or LC3 complex occurs in a context-dependent manner. Step-2: ER-phagy, mediate by receptors FAM134B, SEC62, CCPG1 and TEX264, removes damaged or misfolded protein—loaded ER to preserve proteostasis and secretory capacity, which is important for fibroblast and collagen production and ECM secretion. Golgiphagy, mediated by the YIPF3-YIPF4 receptor complex through LC3/GABARAP binding, maintains Golgi integrity and trafficking, supporting protein modification, membrane delivery, keratinocyte migration and fibroblast ECM secretion. Ribophagy removes selected ribosomes during starvation or cellular stress; the receptor NUFIP1 binds ribosomes and LC3B to promote their lysosomal degradation, enabling amino-acid recycling and adaptation of protein synthesis to the demands of migrating keratinocytes and activated fibroblasts. Step-3. Keratinocyte autophagy supports survival, proliferation, and migration during re-epithelialization. TNF alpha induced autophagy activates CCL2 via the AMPK-BRAF-ERK-AP1 pathway and plays a critical role in keratinocyte differentiation and paracrinally acts on fibroblast differentiation. Step-4: TFEB and TGF-β support fibroblast survival and proliferation and collagen production including the secretion of ECM and collagen. Autophagy also regulates MMP turnover. In conditions of TGF- β driven conditions, excessive autophagy promotes more collagen synthesis, leading to fibrosis. Conversely controlled autophagic conditions promote reduced scarring. Collectively, these processes enhance angiogenesis, re-epithelialization, tissue regeneration, and limit excessive fibrosis and scar formation. Created in BioRender. Rai, P. (2026) https://BioRender.com/rqu1ftp

**Fig. 3:**
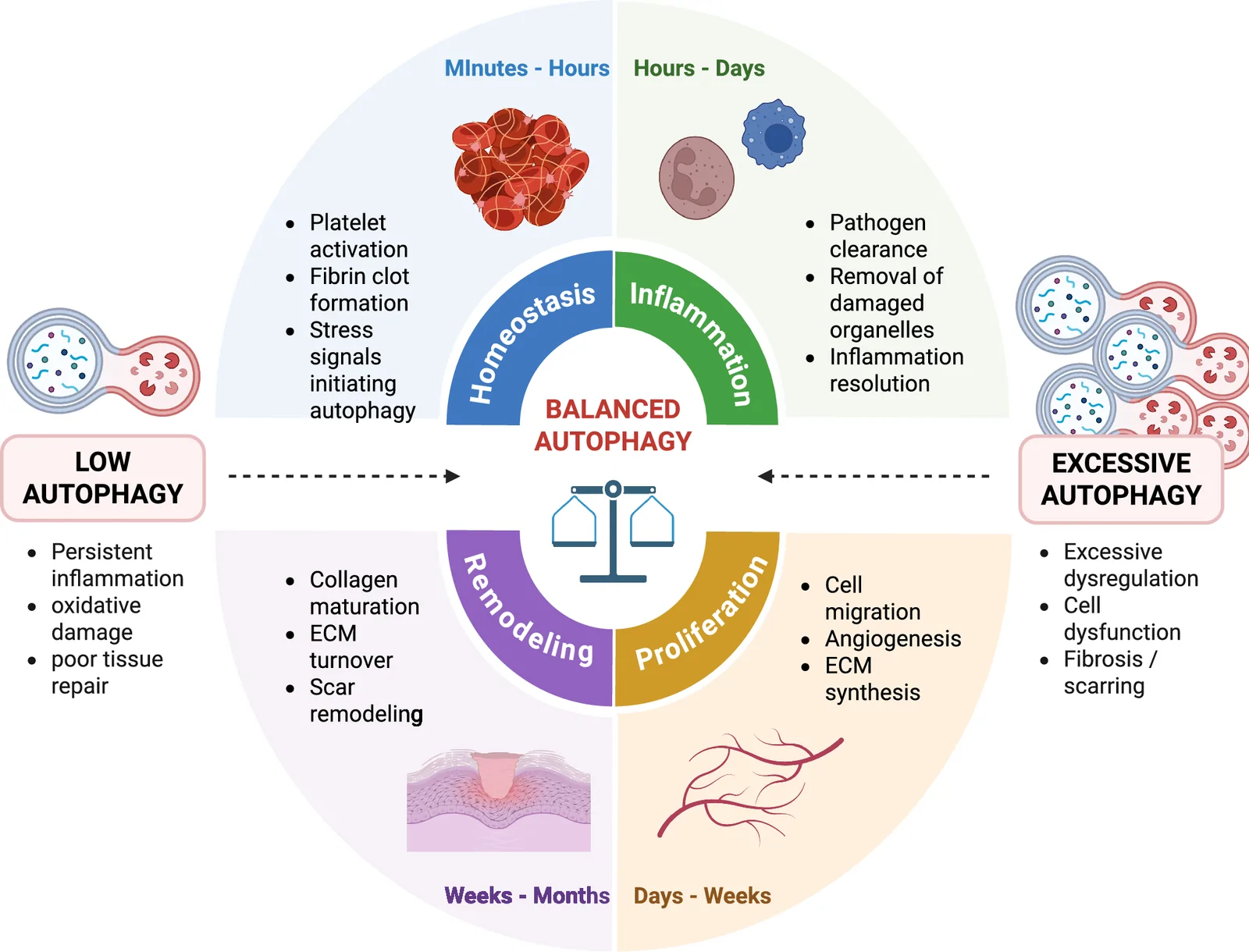
Dynamic role of autophagy balance during wound healing. Autophagy is tightly regulated throughout the hemostatic, inflammatory, proliferative, and remodelling phases of wound repair, contributing to pathogen clearance, angiogenesis, extracellular matrix (ECM) remodelling, and tissue regeneration. Both insufficient and excessive autophagy impair healing outcomes, highlighting the importance of maintaining balanced autophagic activity for effective tissue repair and prevention of fibrosis and chronic inflammation. Created in BioRender. Rai, P. (2026) https://BioRender.com/rqu1ftp

During the proliferative stage of wound healing, autophagy can control fibroblasts and keratinocytes as they differentiate, multiply, and migrate to the wound, which is essential for re-epithelialization and tissue repair (Fig. 2, 3, Tables 1, 2 and 3). Along with these roles, autophagy also helps protect the healing environment by regulating oxidative stress, preventing excessive apoptosis, supports angiogenesis and cell survival [19]. Following injury, hypoxia develops within the wound bed, leading to increased ROS generation. These ROS activate mitogen-activated protein kinase MAPK/p38 and MAPK/JNK signaling pathways, which subsequently promote Bcl-2/adenovirus E1B 19-kDa-interacting protein 3 (BNIP3)-mediated autophagy. In turn, this process facilitates keratinocyte migration and accelerates wound re-epithelialization [9]. In addition, autophagy triggered by hypoxia helps preserve endothelial cell function and drives angiogenesis, which is crucial for restoring blood flow into the injured tissues [50]. Another study conducted by [51] demonstrated the importance of autophagy in keratinocytes by comparing Atg7 deficient (KO) and Atg7 bearing (WT) cells. Cells lacking Atg7 showed lowered resistance to oxidative stress, both intrinsic as well as environmental resulting in DNA damage, disturbed lipid phenotype and cell cycle arrest, all of which point towards premature cellular senescence. Notably the evidence pointing toward hypoxia-driven autophagy promoting keratinocyte migration is assessed by analysing the expression of autophagy related proteins and their surrogate biomarkers(LC3, p62) using immunofluorescence or through western blotting [9]. However these methods reflect the amount of proteins at a single time point and hence do not reflect the presence of autophagic flux and as such, should be used in tandem with autophagy related drugs or fluorescent tagged probes (GFP-LC3 or mRFP/mCherry-GFP-LC3) in future wound studies [52, 53].

**Table 1:** Factors and functions in different stages of wound healing

Stages of wound healing	Key cells and factors	Function	Source
Hemostasis/Coagulation	Platelets	Immediate stop of bleeding through vasoconstriction	[14]
	Fibrin	Formation of a blood clot which serves as the initial provisional matrix for cell migration	[11]
	Growth factors and cytokines	Released from activated platelets to initiate the immune response and attract inflammatory cells	[16]
Inflammation	Neutrophils	First responders and remove bacteria, foreign particles, and damaged tissue Release Reactive Oxygen Species (ROS) and cytotoxic granules and neutrophil extracellular traps (NETs) Release pro-inflammatory cytokines (e.g., TNF-α, IL-1β, IL-6)	[9]
	Macrophage	Arrive after neutrophils, Continue debris clearance and phagocytosis Polarize from pro-inflammatory M1 phenotype (cytotoxic, microbicidal) to pro-repair M2 phenotype (anti-inflammatory) Release growth factors (e.g., VEGF, TGF-β) to stimulate the next phase	[9]
	Mast cell	Degranulation triggers acute inflammation, releasing factors that attract neutrophils Involved in increased vasodilatation and vascular permeability	[16]
Proliferation	Fibroblasts	Migrate into the wound and Proliferate Responsible for granulation tissue formation Synthesis/secretion of ECM components (e.g., collagen, fibronectin, hyaluronic acid, proteoglycans)	[49]
	Myofibroblasts	Facilitate wound contraction to wound edges	[14]
	Keratinocytes	Proliferate and migrate from wound edges to cover the wound, leading to re-epithelialization	[73]
	Endothelial Cells	Form new blood vessels (Angiogenesis/Neovascularization) driven by factors like VEGF and FGF	[19]
Remodelling/Maturation	Fibroblasts/Myofibroblasts	Rearrangement and maturation of the newly synthesized tissue	[12]
	Collagen Type III and Type I	This increases tissue tensile strength (up to 70–80% of original strength)	[9]
	Macrophages/Endothelial Cells	Apoptosis (removal of excess cells) Vascular pruning (regression of excess blood vessels)	[19]
	Matrix Metalloproteinases (MMPs)	Enzymes responsible for degradation and remodelling of collagen and ECM components	[17]

**Table 2:** The autophagic manoeuvring of heterogenous members present in the wound microenvironment have been elucidated in the table below

Stages of wound healing	Cellular components	Key mechanism	Source
Inflammation	Macrophage	AGE-induced autophagy skews polarization toward M1; secretory autophagy released IL-1β/HMGB1 CCL6 suppresses macrophage autophagy to raise M2 levels	[74]
	Neutrophils	Atg5/Atg7 loss significantly reduces degranulation and ROS output; Autophagy induction linked to NET regulation	[9]
	Cytokine	Increased autophagic activity under the effect of cytokines (IL1, IL6, IL10, IL18), TNF and IFNG reaching normal levels at 72 h after thermal burns Increased biomarker levels of LC3 (specifically LC3II and BECN1	[9]
Proliferation	Fibroblast	Atg7 deficient mice demonstrated disrupted TGFB1-induced fibroblast proliferation, migration and myofibroblast transition	[75]
	Myofibroblast	Attenuated ATCA2/α-SMA production in Atg7 knockout mice	[75]
	Collagen	Atg7 knockout mice displayed significantly decreased levels of cross-linked and dense collagen deposition in opposition to their control counterpart	[75]
	Keratinocytes	CCL2 induction via autophagy-AMPK-BRAF-MAPK1/3/ERK-AP1 pathway; Atg5/Atg7 knockdown reduces migration and proliferation	[6]
Remodelling	Endothelial cells	Autophagy via AMPK/Akt/mTOR signalling promotes proliferation, migration and tube formation, sustaining vascular maturation during recovery of heat denatured endothelial cells	[50]
	Fibroblasts/myofibroblasts	Sustained autophagy exhibited activation of mTORC2 and CTGF, thereby driving fibrosis by enhanced differentiation of fibroblasts. Autophagy inhibition stopped the differentiation of myofibroblasts thereby highlighting a critical role in myofibroblast induction	[54]
	ECM	Downregulation of autophagy in the remodelling stage prevents hypertrophic scar formation by degradation of excess ECM components and improving the overall wound site deposition of ECM	[17]

**Table 3:** Current autophagy-targeting therapeutic approaches for wound healing

Compound	Target	Effect on autophagy	Wound healing outcome	Limitations	References
Rapamycin	mTOR	Upregulation	Angiogenesis, re-epithelialization	Immunosuppression	[29, 76]
Metformin	AMPK	Upregulation	Collagen deposition	Poor skin penetration	[24, 59, 77]
Resveratrol	PI3K/AKT/Nrf2	Upregulation	Anti-ferroptosis	Low bioavailability	[25]
Epigallocatechin gallate (EGCG)	AMPK/ULK1	Upregulation	Keratinocyte migration	Limited clinical data	[26]
Curcumin	ROS/autophagy	Upregulation	ECM remodelling	Poor solubility	[64, 65]

### Role of autophagy during the remodelling phase

When wound healing reaches the remodelling stage, autophagy continues to serve as a key regulatory mechanism. In fibroblasts, autophagy is a central regulator of the myofibroblast phenotype: sustained autophagic flux is driven to myofibroblasts by mTORC2 activation and connective tissue growth factor (CTGF) upregulation, along with the acquisition of α-SMA and increased production of COL1A1 and COL3A1 and the formation of stress fibers [54]. This is in turn strengthened by the evaluation of autophagy mediated by transcription factor EB(TFEB), which sustains TGF-β1-induced fibroblast activation and COL 1 release thereby relieving ER stress and reduced apoptosis [55]. Conversely, [56] showed that p53 modulated autophagy can promote autophagic fibroblast apoptosis, thereby limiting hypertrophic scar formation (HSF).. Studies from thermal burn models further emphasize the importance of autophagy in cutaneous wound repair. Keratinocyte growth factor enhances keratinocyte differentiation during both the proliferative and remodelling phases by inducing LC3 expression and activating autophagy through the PI3K–AKT–mTOR pathway. When keratinocytes lack Atg5, they fail to differentiate properly, proving how important autophagy is required for epidermal regeneration [15]. Transcriptomic studies also showed the relation in autophagic influence on human wound repair. Analysis of patients for healing duration of 0, 7, and 14 days, identified 226 differentially expressed genes, included two autophagy-associated genes EIF2AK2 and RB1 that were seen to contribute to the wound healing processes [57]. Eukaryotic translation initiation fcator 2 alpha kinse 2 (EIF2AK2) is involved in both pro- and anti-inflammatory pathways, and RB1 affects innate immune cell differentiation. Validation studies showed that both genes expression increased during healing, indicating a positive regulatory effect [57]. These suggest that autophagy-related regulatory processes can also influence chronic wounds, where they can reduce inflammation, promote angiogenesis, support re-epithelialization, and restore impaired cell function [9, 19].

## Pharmacological modulators of autophagy

Pharmacological modulation of autophagy has emerged as a promising therapeutic strategy for wound healing (Figs. 2 and 3, Tables 1, 2 and 3). By targeting key signaling pathways involved in autophagy regulation, several agents have shown beneficial effects in promoting tissue regeneration and improving healing outcomes in chronic and diabetic wounds [58]. Small molecules modulators of autophagy signaling pathways such as AMPK and mTOR signaling pathways have been found to normalize autophagy balance, induce angiogenesis, improve functions of keratinocytes and fibroblasts, and promote re-epithelialization in preclinical studies [50]. We also performed a comparison of pharmacological agents, natural compounds, stem cell-derived exosomes, and biomaterial-based delivery systems, highlighting their mechanisms of autophagy regulation, biological effects, and therapeutic potential in wound repair (Table 3).

### Rapamycin

Rapamycin, a common autophagy inducer that includes the inhibition of mTORC1, usually accelerates wound healing in preclinical models. Rapamycin gels minimize scar tissue, promote blood vessel growth and re-epithelialization of rodent skin injuries [23]. Similarly, rapamycin-treated mesenchymal stem cells (MSCs) produce exosomes, the application of which in the hydrogel form enhances the healing of diabetic wounds by promoting angiogenesis mediated by VEGF and replacing the (M1) macrophage phenotype [78]. The downside of rapamycin includes producing strong immunosuppressive effects and inducing metabolic adverse effects such as glucose intolerance. In transplant patients, rapamycin has been reported to impair skin repair, so even topical use should be approached with caution to avoid suppressing the inflammation needed for normal healing [29].

### Metformin

Metformin activates AMPK and autophagy. Topical metformin in cream or gel form has been shown to be effective in promoting the closure of diabetic wounds in rats, and it increases autophagy markers such as LC3B and Beclin-1. In a study, a 3 mM metformin gel enhanced collagen deposition and re-epithelialization in diabetic rat wounds through AMPK/mTOR modulation [24, 60]. A systematic review also reported that metformin cream accelerated healing in several rodent models by promoting angiogenesis, reducing inflammatory signaling, and increasing M2 macrophage polarization [59]. Oral metformin has minimal effects on the skin, whereas topical formulations may cause local irritation and require monitoring for systemic absorption. Although metformin influences multiple signaling pathways, its exact mechanism in skin autophagy remains incompletely understood [30, 60].

### Other small molecules

Other small molecules have also shown promise. Trehalose, for example, enhances autophagy through TFEB activation and has been associated with improved wound healing in mice by inducing a transient pro-healing senescent state in fibroblasts through ATG5/7 upregulation. Lithium and spermidine, although not yet tested in skin wounds, have improved autophagy in other tissues and may be worth exploring further [60]. Although evidence has been provided for autophagy activation in innate-immune studies as well as pharmacological activation, it is based on static autophagy marker readouts such as Beclin-1 stabilization, or abundance of LC3 puncta. While these measurements report autophagosome, they cannot differentiate induction of autophagic flux from a block in autophagosome-lysosome fusion or lysosomal degradation, thereby being unable to confirm intact clearance of autophagy [53, 61]. Confirmation of enhancement or restoration of autophagic flux therefore requires flux assays such as the bafilomycin A1 block or fluorescent tagged probes as mentioned in Sect. "Autophagy and wound healing", rather than steady state markers alone [53, 61, 62].

## Phytochemicals and bioactive compounds

### Resveratrol (RSV)

Recent in vitro and in vivo studies have demonstrated that resveratrol exerts significant therapeutic effects in diabetic wound healing. Resveratrol alleviates hyperglycemia-induced inflammation, reduces endothelial cell senescence, and promotes angiogenesis, thereby facilitating tissue repair. Mechanistically, resveratrol modulates the PI3K/AKT/Nrf2 signaling pathway to inhibit ferroptosis, an iron-dependent form of lipid peroxidation-mediated cell death. Consequently, mitochondrial function is preserved, ROS generation and lipid damage are reduced, and cellular proliferation and survival are enhanced [25].

### Epigallocatechin gallate (EGCG)

Another bioactive compound demonstrating promising results in the wound healing domain is epigallocatechin gallate or EGCG. Derived from the plant *Camellia sinensis,* more popularly known as green tea, it has been shownthat EGCG restores impaired autophagy in high glucose-treated keratinocytes, an in vitro model of diabetic wounds. EGCG increased the expression of autophagy-related markers while reducing p62 levels, indicating improved autophagic flux, primarily through activation of the AMPK/ULK1 signaling pathway. Furthermore, EGCG promoted keratinocyte proliferation, migration, and CCL2 secretion, thereby facilitating the recruitment of fibroblasts and immune cells to the wound site. [26, 63]. EGCG also showed autophagy regulation via increased levels of Beclin-1 expression as well as ROS generation. Decreased levels of p62 expression and LC3 transition also aided in the regulation process. Owing to its abundance in the green tea leaves and its antioxidant, anti-inflammatory and anti-microbial properties, it has quickly become one of the promising agents for wound healing in the upcoming years. However, low bioavailability restricts the clinical application of EGCG due to it being unstable under the alkaline conditions of the gastrointestinal tract. Although some positive in vivo and in vitro results have been achieved further human studies need to be conducted to determine whether the same can be replicated in clinical conditions [63].

### Curcumin

Topical curcumin also accelerates wound healing, enhances granulation tissue resulting in a greater amount of collagen deposition and promotes re-epithelialization in mouse models of full-thickness skin defects. It modulated the early inflammatory cytokines and matrix regulators leading to an improved wound structure. Curcumin reduced ROS and enhanced antioxidant enzyme activity in cultured human dermal fibroblasts cultured under oxidative stress. It enhanced cell proliferation, reduced cell senescence and regenerated autophagy as evidenced by enlarged autophagic vacuoles and autophagic-associated proteins [65]. The positive effects were lost when autophagy was inhibited, which demonstrates that the protective effect of curcumin in the presence of oxidative stress is strictly associated with the ability of the latter to induce autophagy. It is also indicated in the same study that curcumin may be useful in the prevention of fibrosis and scars. It could enhance collagen remodelling and minimize the development of unhealthy scars through the modulation of fibroblast autophagy and oxidative stress [65].

### Other phytochemicals and their use in biomaterials

Novel combinations such as quercetin and curcumin are under investigation for their impact on fibroblast migration and closing fibroblast monolayer wounds. Quercetin alone accelerates the healing process and the combination of the two presented better antioxidant and antimicrobial effects than curcumin alone [66]. Flavonoids including catechin, epicatechin, quercetin, and luteolin have demonstrated potential wound-healing potential. They mediate through various mechanisms in addition to autophagy, forming a large beneficial action [67]. These are increasingly being incorporated with bioactive compounds into tissue-engineering scaffolds, hydrogel or nanofiber to enhance their low bioavailability and skin penetration. The local concentrations of drugs can be sustained by controlled release of these materials and reduce systemic exposure, as well as provide a favorable environment that initiates autophagy and allow cells to survive the stresses present in the wound sites [68].

## Cell-based and biomaterial-based strategies in wound healing

### Mesenchymal Stem Cell (MSC) exosomes

Adipose-Derived Stem Cells (ADSCs) or Human Umbilical Cord Mesenchymal Stem Cells (hUCMSCs) exosomes have the ability to transfer miRNA and proteins, which influence autophagy in the recipient cells. For instance, rapamycin-primed exosomes of MSCs (RAPA-sEVs) accelerated the wound healing in diabetic injury. They suppressed the growth of macrophages and the secretion of cytokines, such as TNF-α and IL-1β, along with enhancing the motility and tube formation of endothelial cells [27]. This interaction depended on the PI3K/AKT pathway implicating the role of autophagy. Autophagy gene overexpression in keratinocytes or fibroblasts is yet to be tested. There are no clinical stem-cell products which specially influence autophagy [17].

### Scaffolds and gels

Biomaterial dressings based on tissue engineering are also being engineered to regulate autophagy directly at the wound microenvironment providing a direct means of controlling wound healing in skin wounds, including burns, cuts, diabetic ulcers and pressure sores. A key strategy is the use of hydrogel e.g., zwitterionic sulfobetaine methacrylate hydrogels. These are capable of enhancing fast wound healing by preventing major negative autophagy behavioral antagonists (PI3K/AKT/mTOR), leading to increased collagen deposition, re-epithelialization and recovery of normal extracellular matrix proteins like laminin and fibronectin [28]. Thermosensitive injectable hydrogels with dual-functional agents (zinc-metformin) have demonstrated satisfactory healing performance as zinc aids in the growth of keratinocytes and the metformin facilitates the initiation of autophagy. This combination upregulates the expression of LC3 at the wound site, reduces inflammatory cytokines and accelerates full-thickness skin repair [68]. Certain scaffolds specifically inhibit autophagy in the case of chronic overactivation being detrimental, such as poly-lactic-co-glycolic acid (PLGA) microspheres. These expressing (PDGF) which inhibit excessive autophagy in endothelial cells and fibroblasts causing a burst of growth signaling to stimulate angiogenesis and granulation tissue development [69]. Electrospun chitosan/polyvinylpyrrolidone (CS/PVP) nanofibers provide a high-surface-area dressing that closely mimics the native extracellular matrix, thereby supporting cell adhesion, proliferation, and tissue regeneration. Furthermore, incorporation of phytochemical antioxidants such as dihydromyricetin (DHM) into CS/PVP nanofibers has been shown to enhance the expression of autophagy-associated proteins, including LC3-II, ATG5, and ATG7, while simultaneously suppressing inflammatory signaling pathways, ultimately promoting diabetic wound healing [70].

Notably, tissue-engineered systems enable localized and sustained delivery of autophagy modulators with minimal systemic toxicity and wound-bed targeting. More sophisticated techniques involve the use of both biomaterials and stem cells, including mesenchymal stem cell-encased hydrogels preinduced with autophagy stimulators like rapamycin, which promotes MSC survival, angiogenic factor release and macrophage polarization to the healing phenotype [71]. Altogether, the scaffold-based approaches indicate that the time and localization of autophagy induction are an effective method to induce the high-quality skin repair, minimize scarring, and enhance vascularization in both acute and chronic wound models [9].

## Challenges and future perspectives

Despite the growth of interest in targeting autophagy for wound repair, several biological and translational challenges remain as a concern. The role of autophagy in wound healing varies with the stage of the process, cell type, and wound etiology. While autophagy induction promotes re-epithelialization, angiogenesis, and collagen maturation in acute wounds [6], chronic wounds like diabetic ulcers, often exhibit dysregulated autophagy associated with persistent oxidative stress and inflammation. Thus, therapeutic strategies require precise regulation rather than uniform activation or inhibition of autophagy [17].

One of the major limitations are translation into clinical practice which is limited by the poor physicochemical properties of many autophagy-modulating compounds, including rapamycin, metformin, resveratrol, curcumin, and EGCG. Their low solubility, limited skin penetration, instability, and unpredictable pharmacokinetics necessitate advanced delivery systems such as hydrogels, liposomes, nanocarriers, microneedles, and stimulus-responsive biomaterials [71]. Moreover, many currently available modulators lack pathway specificity and may simultaneously influence inflammation, fibroblast proliferation, keratinocyte migration, and angiogenesis, increasing the risk of off-target effects [20]. Systemic administration may also lead to immunosuppression, tissue toxicity, or delayed wound closure [29].

Another major limitation is the scarcity of robust clinical evidence. Most studies rely on rodent models, diabetic mouse wounds, ex vivo human skin, or small preclinical investigations, with relatively few well-controlled clinical trials available to validate therapeutic efficacy in patients [3]. In addition, challenges associated with biomaterial reproducibility, sterility, biodegradability, manufacturing costs, and batch-to-batch variability of natural products continue to hinder clinical translation [59]. Future research should focus on developing cell- and stage-specific autophagy modulators, particularly those targeting selective autophagy pathways such as mitophagy, xenophagy, and ER-phagy. Combining these agents with smart biomaterials capable of controlled, localized, and sequential drug release may enable dynamic regulation of autophagy throughout the healing process. Such strategies have the potential to maximize tissue regeneration while minimizing fibrosis and other adverse effects, ultimately facilitating the clinical translation of autophagy-based wound therapies,

## Discussion

Growing evidence suggests that autophagy is not just a cellular recycling mechanism but an important regulator of multiple events that determine successful wound repair [72]. Autophagy integrates metabolic adaptation, inflammatory signaling, and tissue remodelling to maintain cellular homeostasis throughout the healing process. Its influence extends across all stages of wound healing, from regulating the early inflammatory response to supporting keratinocyte migration, angiogenesis, extracellular matrix remodelling, and scar maturation [9, 19]. Nevertheless, the present literature is insufficient to conclude that autophagy plays an unambiguously positive or negative role in the process. Its effect depends on wound pathology, cell type, level of activation, and phase of repair. The mentioned context dependency might explain the contradictory data observed in acute wounds, diabetic ulcers, infection-induced wounds, and thermal injury models.

Controlled activation of autophagy generally promotes tissue repair by limiting oxidative stress, maintaining mitochondrial integrity, and preventing excessive inflammation. However, persistent or dysregulated autophagy, particularly in chronic wounds such as diabetic ulcers, may contribute to impaired healing. These observations indicate that therapeutic modulation of autophagy should consider the timing, cellular context, and wound microenvironment rather than relying on generalized activation or inhibition [17, 18]. It should be noted that increased levels of autophagy markers should not be considered proof of increased autophagic activity per se. For example, increased amounts of LC3-II may point either to increased autophagosome formation or to defective autophagosomal degradation due to impairment of lysosomal functions. Changes in Beclin-1 and p62/SQSTM1 do not represent sufficient information about autophagy itself. Future research on wound healing should differentiate between autophagy activation and autophagic flux using LC3 processing, p62 degradation, lysosomal function testing, and lysosomal degradation inhibition. To this end, autophagic flux assays particularly the bafilomycin A1(lysosomal inhibition) block assay and the tandem MRFP-GFP-LC3 reporter, together with the LC3-II turnover and p62/SQSTM1 degradation as standardized flux readouts for wound healing research are a viable and standardized set of techniques that can be used in combination with the aforementioned static marker proteins. However, these probes are unsuitable for the clinical analysis of autophagy in the tissue sample of patients. Hence there is no conclusive one-method-for-all approach that can be taken. Instead, multiple assays can be used in tandem for better enhancement and assessment of autophagy and autophagic flux [9, 53, 62].

Recent studies have also expanded the translational potential of autophagy-based therapies. Pharmacological agents, phytochemicals, stem cell-derived exosomes, and biomaterial-based delivery systems have all demonstrated the ability to regulate autophagy while improving angiogenesis, re-epithelialization, collagen deposition, and immune modulation in preclinical models [6, 15]. Nevertheless, most evidence remains limited to experimental studies, highlighting the need for well-designed clinical trials to determine the safety, efficacy, and optimal therapeutic window of autophagy-targeting interventions. Further complicating the interpretation of the results from preclinical studies is the presence of considerable variability between different types of wound models in terms of the available oxygen, vascularization, microbiota, cellular content, and metabolic stress. Therefore, while therapy can be effective in the treatment of a clean acute wound, it does not mean that it would have the same effect in case of chronic diabetic and infected wounds. For future research, it is recommended to differentiate results for each specific type of wound, rather than considering all skin injuries equal from a biological perspective. For future translation into clinical practice, there is a need for reliable biomarkers that would help differentiate those wounds for which it is desirable to induce autophagy, from those that need to suppress or normalize autophagy.

To conclude, the current evidence base suggests that autophagy holds considerable potential for a new therapeutic approach in wound healing. However, the main goal of therapy should not be the random activation or deactivation of autophagy processes. Rather it is important to achieve restoration of the normal autophagic flux in the appropriate type of cells, wound area, and at the proper stage of healing.

Future strategies that combine selective modulation of autophagy with advanced drug delivery systems and biomaterials may provide more precise and effective approaches for managing chronic and non-healing wounds. In particular, smart biomaterials capable of releasing drugs locally, continuously, or sequentially can prove valuable; however, the universal strategy of early stimulation and later inhibition of autophagy processes cannot be applied in all cases.

There are several key issues that need to be addressed before moving towards clinical application of this therapeutic approach. It is not clear whether the primary problem is the failure of initiation of autophagy or lysosomal clearance in various chronic wounds, which cells to target during each phase of the healing process, and whether selective pathways such as mitophagy, xenophagy, and ER-phagy are superior to the manipulation of general autophagy per se. To answer these questions through systematic measurement of fluxes and by using clinically relevant animal and human models will be crucial for establishing whether autophagy-based treatments can provide any benefit to patients with chronic wounds.

## Conclusion

Autophagy is no longer viewed as merely a degradative pathway but as a master regulator coordinating inflammation, metabolism, angiogenesis, extracellular matrix remodelling, and tissue regeneration. Emerging evidence suggests that spatially and temporally controlled modulation of autophagy may transform chronic non-healing wounds into regenerative environments. Future therapeutic strategies should therefore focus on cell-specific and stage-dependent regulation of autophagy combined with advanced biomaterials and precision drug delivery to maximize repair while minimizing fibrosis.

## Supplementary Information

Below is the link to the electronic supplementary material.

Supplementary file1 (PDF 408 KB)

Supplementary file2 (PDF 693 KB)

Supplementary file3 (PDF 417 KB)

## Data Availability

All materials about this manuscript are available in the draft; no new data was generated.
